# Bigger Is Not Always Better: Females Prefer Males of Mean Body Size in *Philautus odontotarsus*

**DOI:** 10.1371/journal.pone.0149879

**Published:** 2016-02-22

**Authors:** Bicheng Zhu, Jichao Wang, Longhui Zhao, Zhixin Sun, Steven E. Brauth, Yezhong Tang, Jianguo Cui

**Affiliations:** 1 Chengdu Institute of Biology, Chinese Academy of Sciences, Chengdu, Sichuan, China; 2 Department of Biology, Hainan Normal University, Haikou, Hainan, China; 3 Department of Psychology, University of Maryland, College Park, Maryland, 20742, United States of America; University of Thessaly, GREECE

## Abstract

Most species are believed to evolve larger body sizes over evolutionary time. Previous studies have suggested that sexual selection, through male-male competition and female choice, favors larger males. However, there is little evidence of selection against large size. The female serrate-legged small treefrogs (*Philautus odontotarsus*) must carry passive males from leks to breeding grounds over relatively long distances after amplexus to find a suitable place to lay eggs. The costs of large male size may therefore decrease mating success due to reduced agility and/or higher energy requirements. Thus, we hypothesized that selection would *not* favor larger males in *P*. *odontotarsus*. Females can assess male body size on the basis of the dominant frequency of male calls in frogs. To assess female *P*. *odontotarsus* preferences for a potential mate’s body size, male calls of high, average and low dominant frequency were played back to the females in phonotaxis experiments. Results showed that most females prefer the advertisement call with average dominant frequency. In addition, we compared the body mass distribution of amplectant males with that of single males in nature. The body masses of amplectant males are more narrowly distributed in the intermediate range than that of single males. The phonotaxis results and the data of actual female preferences in the field show that females strongly prefer potential mates of mean body sizes, consistent with the view that, in this species at least, larger males are not always perceived as better by females. In the present study, *P*. *odontotarsus* provides an example of an amphibian species in which large size does not have an advantage in mating success for males. Instead, our results provide evidences that stabilizing selection favors the optimal intermediate size of males.

## Introduction

Body size is crucial to many physiological and ecological processes [1–3]. Lineages are believed to evolve larger body sizes over evolutionary time (Cope's rule) [3–5]. There is substantial empirical evidence showing that in many species sexual selection (male-male competition or female choice) in males favors larger body size due to the greater mating and reproductive success of large males [6]. Previous studies in anuran species have shown that large males can defeat small males in aggressive encounters, displace small males from amplexus or from territories, and often exhibit greater reproductive success [7–11]. In addition to male-male competition, female *Physalaemus pustulosus* preferentially chooses large males [12–13], thus supporting the idea that sexual selection in anurans, through male-male competition, female choice, or both, favors large males in a number of species.

Despite the advantages of large size, however, sexual selection usually has not led to larger males in anurans, implying that other selective forces against large body size are in play. The equilibrium view is that selection for large body size is eventually counterbalanced by opposing selective forces, primarily viability selection [6, 14–18]. From anurans specifically, Woolbright (1983) proposed that male body size was limited by a suite of energetic constraints associated with reproduction [19]. For example, large males may prove too great a burden for females during amplexus and may not be able to quickly escape from predation due to reduced agility or high energy requirements [18, 20]. As a particular example, Dziminski et al. (2010) reported that there was a negative relationship between sperm viability and male body size [21]. Thus in such cases selection would not favor females preferring the largest males since such choices would decrease the direct benefit to the females [22]. The problem then becomes one of understanding how sexual selection acts to maintain and stabilize male body size when relatively large male body sizes may decrease female fitness. To test the above hypothesis, the present study investigated female choice in the serrate-legged small treefrog, *P*. *odontotarsus*, a species in which females must carry passive males to breeding sites.

The serrate-legged small treefrog is a typical tropical anuran species in which competition between males for potential mates is intense. The serrate-legged small treefrog, *P*. *odontotarsus*, exhibits distinct sexual dimorphism in body size between males and females. Furthermore, our preliminary experiment showed that, after amplexus, female *P*. *odontotarsus* must carry male mates for long distances (26.6 ± 7.57 m, n = 11) from leks to breeding grounds in order to find a suitable place to lay eggs. The whole process of egg laying lasts about 4 hours. Compared with carrying smaller males, females who mate with larger males incur higher energy costs and predation risks. Hence, we hypothesized that female *P*. *odontotarsus* would prefer moderately sized males rather than the largest male available due to the combined selection pressures of male-male competition (favoring larger male body sizes) and the female’s energetic burden (favoring smaller male body sizes).

Many studies have demonstrated that, for anurans, both males and females can obtain important information about the males’ body size from their calls [12, 23–25]. A previous study of our group has demonstrated that there is a significant negative correlation between the dominant frequency of male calls and body size in *P*. *odontotarsus* (S1 Fig). To test if female *P*. *odontotarsus* would prefer moderately sized males rather than the largest males, calls of high, average and low dominant frequencies were played back to the females in phonotaxis experiments. Additionally, the body size of the female subjects was measured in order to determine if mate choice was correlated with female body size in this species.

## Materials and Methods

### Study site

Our experiments were conducted in the Mt. Diaoluo National Nature Reserve in Hainan, China (18.44°N and 109.52°E, elevation of 933 m) from July to September, 2014. The serrate-legged small treefrog (*P*. *odontotarsus*) is a typical tropical species that lives in moist shrubland or intermittent freshwater marshes, and males have a relatively small body size and produce complex calls to attract females during the night from February to September. The local air temperature was 17~24°C during this period. Relative humidity usually approached 100% from 18:30 h to 06:30 h, during which calling occurs.

### Acoustic stimuli

**Fig 1:**
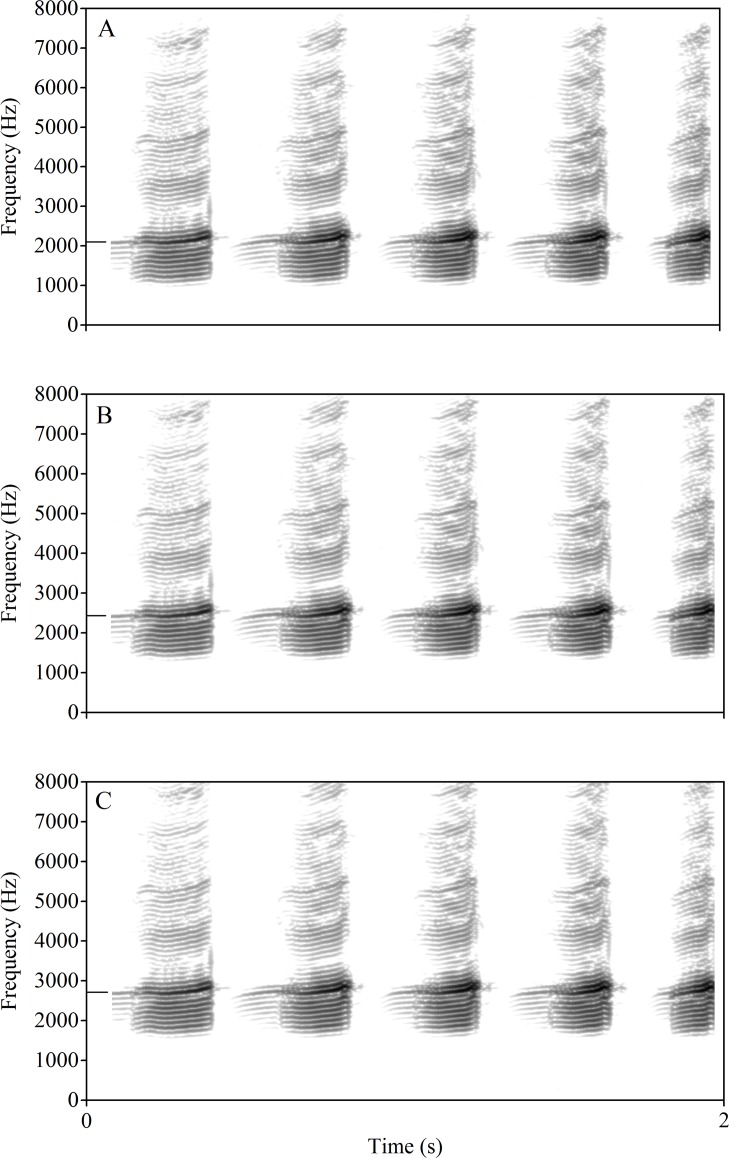
The stimulus calls with different dominant frequencies of *P*. *odontotarsus*. The FFT (fast Fourier transform) frame is 1024. The dominant frequency of the stimulus is marked with a short black bar: (A) low, (B) average and (C) high dominant frequency.

Three stimuli were synthesized using Avisoft SAS-Lab Pro (Avisoft Bioacoustics, Berlin), based on the advertisement calls of male serrate-legged small treefrogs which have been shown to be attractive to females (unpublished data). The dominant frequency of the three stimuli were 2110 Hz representing low frequency calls (L: the lowest dominant frequency in the calls of 62 males), 2713 Hz representing high frequency calls (H: the highest dominant frequency in the calls of 62 males) and 2445 Hz representing average frequency calls (A: the average dominant frequency in the calls of 62 males) (Fig 1), respectively, while the temporal characters remained unchanged. Because the call dominant frequencies are associated with male body masses, the H, A and L dominant frequencies represent smaller, average and larger male body sizes, respectively. Stimulus pairs were constructed as follows: (1) L versus H, (2) L versus A, (3) A versus H.

### Phonotaxis experiments

Two-speaker phonotaxis tests were performed with all stimulus pairs: (1) L-H, (2) L-A, (3) A-H, which were presented to females in a randomized sequence, between 20:00 to 03:00 (Temperature: 18.8~23.2°C, Relative Humidity: 80.9~99.9%). On each trial, one female was tested using a single stimulus pair. Across trials all females were tested using all three stimulus pairs.

We placed each female subject in the center of a sound-attenuating chamber [2.2 (L) × 1.5(W) m], while the stimuli pairs were broadcast from two portable field speakers (SME-AFS, Saul Mineroff Electronics, Elmont, NY, USA) placed equidistantly from the opposite ends of the chamber. We broadcast the test stimuli antiphonally such that the peak amplitude of each test call at the center of the arena was 80 dB SPL (re 20 μPa). The peak sound pressure levels (SPLs) of each test call were adjusted (A-weighted) with a sound level meter (AWA 6291, Hangzhou Aihua Instruments Co.) at the center of the arena.

Females were scored as having made a choice when they approached either speaker within 20 cm without simply following the wall. Females who were motionless for 5 minutes at the release point or remained motionless for any 2 min time period after exiting the release point, or who roamed the arena for more than 10 min without approaching a speaker, were not recorded as having made a choice. The behavior of the females was observed using a wide angle lens video system equipped with an infrared light source. Each frog was given a unique toe-clip number prior to being returned to the environment in order to prevent retesting. Frogs were captured by hand, and the body mass, snout-vent length (SVL) and head width were measured after phonotaxis experiments were completed.

### Analysis and statistics

Data were statistically analyzed using Sigmaplot 11.0 software (Systat Software Inc., Chicago, USA). The binomial test was used to evaluate the phonotaxis data for females in response to male calls with different dominant frequencies. To test if female preferences are related to the female body size, the *K-means* algorithm was used to divide the female body size into three grades. The Chi-square test was used to test the proportion differences between the body size distribution of amplectant males and single males in the field. A significance level of 95% (α = 0.05) was set for all the tests.

### Ethics statement

All applicable international, national, and/or institutional guidelines for the care and use of animals were followed. All procedures performed in studies involving animals were approved by the Animal Care and Use Committee of Chengdu Institute of Biology, CAS (CIB2014031008). This work was conducted with the permission of the Management Office of the Mt. Diaoluo Nature Reserve. This article does not contain any studies with human participants performed by any of the authors.

## Results

### Female phonotaxis responses

**Fig 2:**
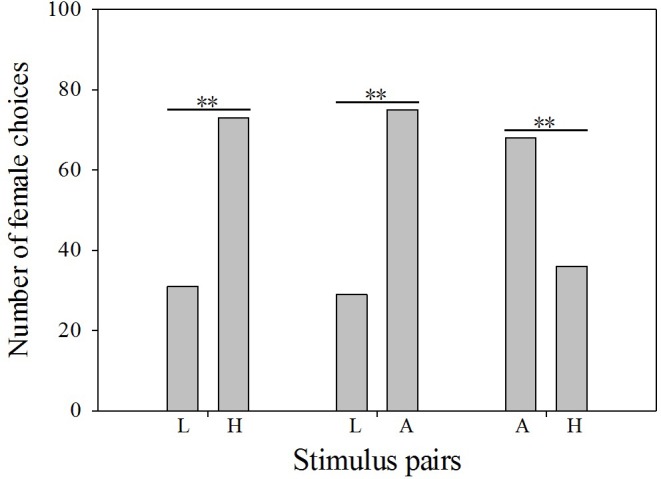
Phonotaxis data for female *P*. *odontotarsus* in response to male calls with different dominant frequencies. L: low dominant frequency call; A: average dominant frequency call; H: high dominant frequency call. Binomial test, **P < 0.01.

In total, there were 125 females utilized in the phonotaxis tests. Some females did not behaviorally indicate a preference (21 females). Accordingly, we obtained 104 samples of female choice behavior. Most females preferred the advertisement call with average dominant frequency over the calls with high (X^2^ = 9.8, P = 0.002, df = 1, n = 104, Fig 2) or low dominant frequency (X^2^ = 20.3, P < 0.001, df = 1, n = 104, Fig 2). Compared with low dominant frequency calls, females preferred calls with high dominant frequency (X^2^ = 20.3, P < 0.001, df = 1, n = 104, Fig 2).

**Fig 3:**
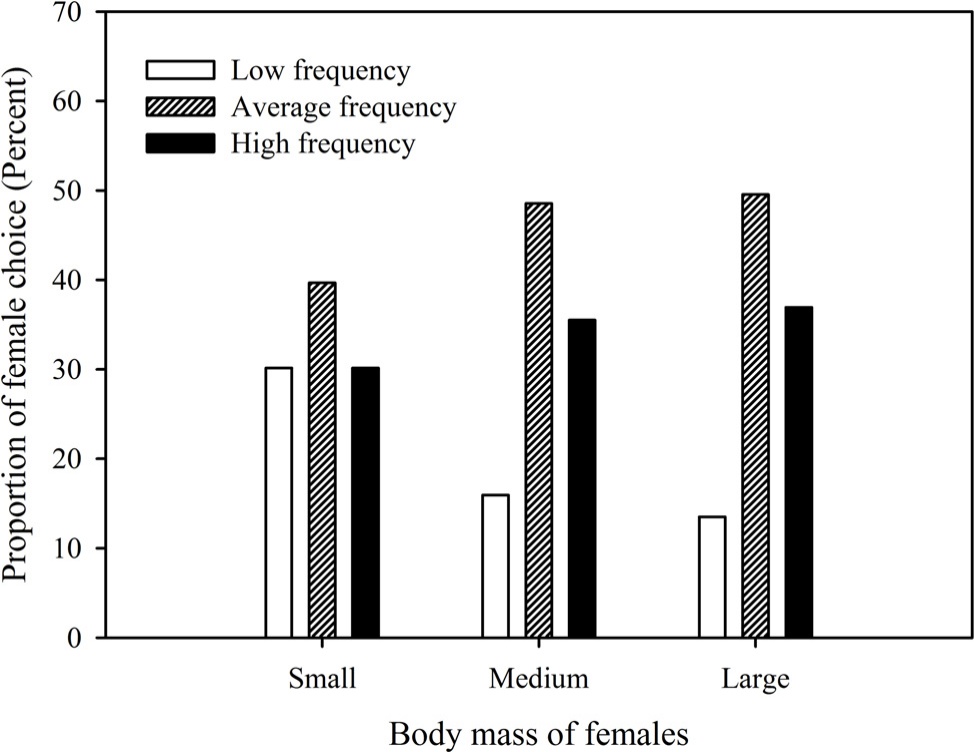
Phonotaxis data for female *P*. *odontotarsus* with different body masses. S: small body mass (S ≤ 6.344g); M: medium body mass (6.348g ≤ M < 7.689g); L: large body mass (L ≥ 7.703). *K-means* algorithm was used to divide the body sizes of females into three grades.

To test if the preference of females is related to their own body size, the *K-means* algorithm was used to divide females into three grades (Small, medium and large) based on female body size (Fig 3). Given that body mass is one of the most important indicators of body size, significantly positively correlated with snout-vent length (*r* = 0.7, P < 0.001, df = 1, *n* = 62) and head width (*r* = 0.74, P < 0.001, df = 1, *n* = 62), we chose body mass alone as an index to simplify the analysis. The results showed that while all females preferred calls of average frequency, females with large and medium masses preferred the high dominant frequency calls over low dominant frequency calls (41 vs. 15, X^2^ = 10.4, P = 0.001, df = 1, large females; 49 vs. 22, X^2^ = 7.7, P = 0.006, df = 1, medium females), while small mass females did not prefer calls of high dominant frequency over those of low dominant frequency (19 vs. 19) (Fig 3). However, the ratio of females preferring low dominant frequency calls in the small body size group (30.2%) was higher than in both the medium body size group (15.9%) (X^2^ = 4.3, P = 0.039, df = 1) and large body size group (13.5%) (X^2^ = 5.8, P = 0.016, df = 1, Fig 3).

### Body size distributions of amplectant males and single males in the field

**Fig 4:**
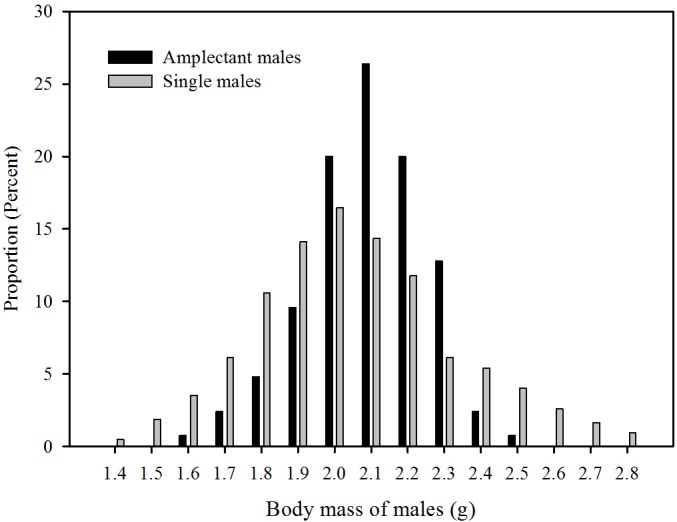
Body size distribution of amplectant males and single males in the field. (n = 425 for single males, n = 121 for amplectant males).

To access the potential influence of female preference on the distribution of male body mass in the natural population, the body masses of 425 single males in the natural population were measured and divided into 15 grades using 0.1 gram as a class interval (Fig 4). The results showed that most males exhibit intermediate (62.8% in the 1.9–2.3 gram range) rather than small (22.6% in the 1.4–1.8 gram range) or large body mass (14.6% in the 2.4–2.8 gram range). To test whether the results of the phonotaxis experiments were in conformity with actual female preferences in the field, we measured the body mass of males in amplectant pairs (n = 121) and compared the body mass distribution of these males with that of single males in nature using variance analysis. The variance of body size for amplectant males (0.027) is smaller than that of single males (0.062), indicated that the body size of amplectant males is more centralized than that of single males. The proportion of individuals in the intermediate range (1.9–2.3 gram) is significantly higher in paired males (88.4%) than that of the single males (62.8%) (X^2^ = 4.5, P = 0.035, df = 1), while the proportions of individuals in the small mass group (1.4–1.8 gram) (X^2^ = 7.3, P = 0.007, df = 1) and large mass group (2.4–2.8 gram) (X^2^ = 8.0, P = 0.005, df = 1) were significantly lower for paired males than for that of the single males (Fig 4).

## Discussion

There are ample evidences that, in anurans, larger males have an advantage in both male-male competition and female choice [6, 26–27]. Larger males usually defeat smaller ones [7–11, 15, 28–30] and can fertilize more eggs [31], while females often prefer relatively larger males [12, 32]. However, while the evidence for fecundity and sexual selection favoring larger body size is overwhelming, evidence of the disadvantages of large body size is relatively scant in the literature, even though these factors are crucial for explaining why the earth is not mainly populated with gigantic organisms [18, 33]. As a particular example, Tammaru et al. found that there was no evidence of costs for being large in females of *Orgyia* spp. (Lepidoptera, Lymantriidae), because none of the fitness components studied was negatively correlation with female body size in *Orgyia* spp. [34].

Insofar as the dominant frequency of male calls is negatively correlated with body mass in *P*. *odontotarsus*, relatively higher dominant frequency values represent the signals produced by relatively smaller males. Since the results of the present study show that females strongly prefer male advertisement calls of average dominant frequency, these results imply that female *P*. *odontotarsus* is biased to choose males with mean body masses. Large males may prove too great burdens for females during amplexus and may not be able to quickly escape from predation as well. Thus in such cases selection would not favor females preferring the largest males [35]. The trade-off between the effects of male-male competition and fecundity advantage which promotes larger male body size and the energetic burden large male body size would pose to females, act jointly to reduce the advantage that large male body size would provide for fitness. As a result, females have apparently evolved to prefer mates of mean body size.

We also found that in their natural environment most males have medium body mass (62.8% in the range of 1.9–2.3 gram) rather than small (22.6% in the 1.4–1.8 gram range) or larger body mass (14.6% in the 2.4–2.8 gram range), and that the body masses of amplectant males are more narrowly distributed than that of single males with a greater proportion of amplectant males in the medium body mass range compared to that of single males (Fig 4). These results are consistent with the results of the phonotaxis experiments showing that females prefer the calls of potential mates with mean body mass.

Previous studies have suggested that sexual selection which favors large body size, along with other selective forces against large body size, such as female perceptual limitations and/or energetic constraints [18, 35–36], as well as the viability costs of long development, fast growth, reduced agility or increased detectability [18], maintain the stabilization of body size in natural populations. Our results show that female choice might limit increases in male body size and maintain male body size within a stable range in serrate-legged treefrogs. According to the evolutionary stable strategy (ESS) theory, for the individual, the best strategy depends on the behaviors performed by most members of the species [37–38]. In the present study, most male frogs possess moderate body mass and females strongly prefer males with mean body mass both in phonotaxis tests and in the field, consistent with the evolutionary stable strategy (ESS) theory, although factors such as viability selection, predation, or other stabilizing mechanisms may also function in maintaining intermediate males in the population.

It is intriguing that relatively large and medium females prefer small males over large males, and that the ratio of the females in the small body size group who prefer large males (30.2%) is higher than the proportion of females preferring large males in the medium body size group (15.9%) and in the large body size group (13.5%, Fig 3). One possible explanation for these preferences is that such choices tend to increase female fitness by increasing the likelihood that male offspring will be of intermediate body size since body mass is heritable [39–40]. Thus, in order to increase the likelihood that offspring would be of average size, large females would be expected to mate with small rather than large males, while the small females should avoid mating with small males.

Gress et al. reported that female yellow dung fly, *Scathophaga stercoraria* (Diptera: Scathophagidae) appeared to preferentially mate with large males on dung, but small males at foraging sites [41]. In the present study, we found that small male *P*. *odontotarsus* were more attractive to females than large males although not as attractive as those of intermediate body size. During amplexus, female *P*. *odontotarsus*, must carry their mates relatively long distances. Therefore, females who mate with large males incur higher energy costs and predation risks. This is consistent with the result that relatively large and medium sized females prefer small males over large males, which would decrease the energy costs and increase the likelihood that male offspring will be of average size. The relatively small females did not prefer small males which might reflect a trade-off between the energetic costs small females would incur in carrying a large male and the reduction in fitness which small females would experience by mating with small males due to the increased likelihood that male offspring would be too small.

In most anuran species males are smaller than females [19] and no single explanation has been proposed to explain this pattern. Males and females are often subjected to different selection pressures, which can result in sexual size dimorphism [33, 42]. Some trade-offs may cause selection against large size only in males. For example, in the present case large male size may increase the burden for females during long copulatory periods, which may result in female-biased sexual size dimorphism [43–44]. For most anuran species, as with *P*. *odontotarsus*, the females must carry male mates from leks to breeding grounds to lay eggs. It is unclear if the mechanism maintaining male body size within a relatively small range is similar to that of *P*. *odontotarsus*. This possibility awaits testing in more species.

Classic sexual selection theory proposes that female choice may drive male sexual signaling or body size to become more exaggerated. Here we show that female choice can act to constrain variation in male body size so that most members of the population exhibit moderate body mass. These results indicate that female choice may not always lead to larger and more extravagant male traits and for female choice bigger males are not always perceived as better. In present study, *P*. *odontotarsus* provides an example of an amphibian species in which large size does not have an advantage in mating success for males. Instead, our result provides evidence that stabilizing selection favors the optimal intermediate size of males.

## Supporting Information

S1 Fig Relationships between body mass and dominant frequency of calls in *P*. *odontotarsus*. (PDF)
