# Supplementary figures and images for: Bigger Is Not Always Better: Females Prefer Males of Mean Body Size in Philautus odontotarsus
Source: PLoS One. 2016 Feb 22;11(2):e0149879. doi: 10.1371/journal.pone.0149879 (PMC4762700; doi:10.1371/journal.pone.0149879)

*odontotarsus*

*odontotarsus*

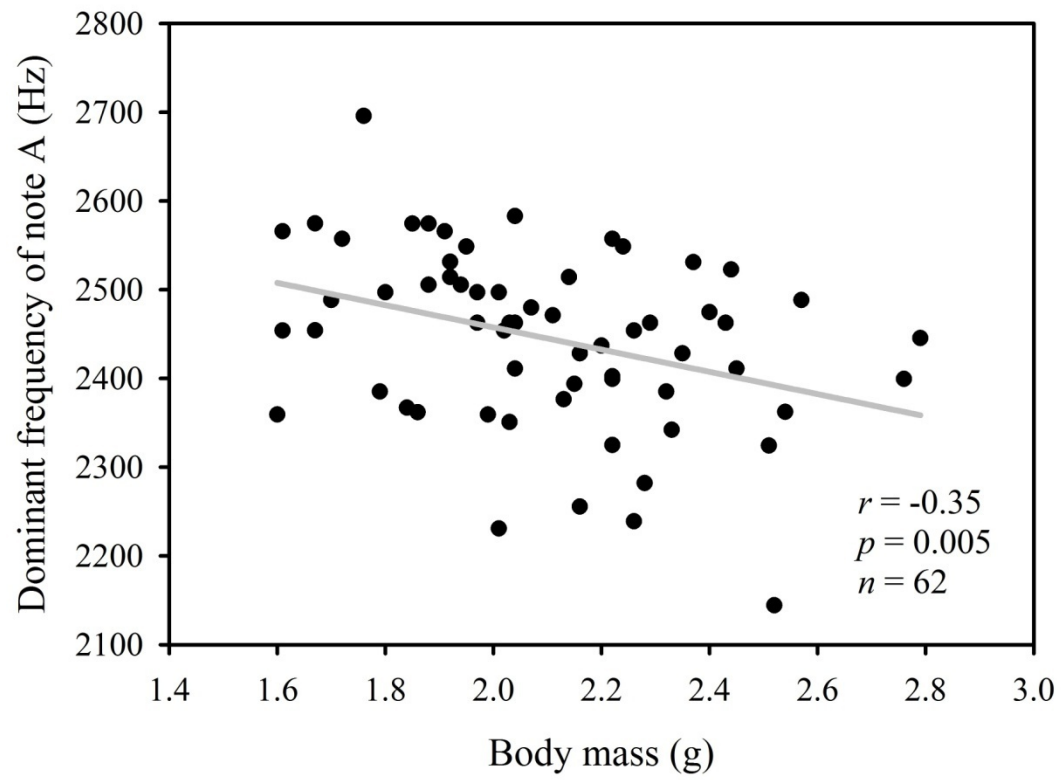

Supplement: S1 Fig — (PDF) [file pone.0149879.s001.pdf]
